# Mothers’ Facebook posts about infant health: findings from the Grow2Gether study

**DOI:** 10.1186/s12887-018-1315-4

**Published:** 2018-10-31

**Authors:** Stacey Kallem, Rachel S. Gruver, Senbagam Virudachalam, Alexander G. Fiks

**Affiliations:** 1 Perelman School of Medicine, University of Pennsylvania, Philadelphia, PA USA; 2 Divison of General Pediatrics, Children’s Hospital of Philadelphia, 2716 South Street, Philadelphia, PA 19146 USA; 3 PolicyLab, Children’s Hospital of Philadelphia, 2716 South Street, Room 10-323, Philadelphia, PA 19146 USA; 4 Leonard Davis Institute of Health Economics, University of Pennsylvania, Philadelphia, PA USA; 5 Center for Pediatric Clinical Effectiveness, Children’s Hospital of Philadelphia, 2716 South Street, Room 10-471, Philadelphia, PA 19146 USA

**Keywords:** Social media, Parenting, Underserved, Infant care, Recommendations, Anticipatory guidance

## Abstract

**Background:**

Social media is a common way for mothers to seek advice about their infants. However, little is known about how low-income urban mothers use social media to obtain infant health information and whether this information is consistent with expert pediatric recommendations.

**Objectives:**

(1) identify the types of health questions asked by low-income mothers of infants in a social media parenting group; (2) describe whether peer answers are consistent with or contradict AAP guidelines; (3) identify the practices that mothers post about that are inconsistent with AAP guidelines.

**Methods:**

Forty-three low-income mothers were enrolled in Grow2Gether, a private Facebook group intervention focused on infant care and moderated by a psychologist. All health questions posted by mothers were coded thematically; answers to questions from the group were assessed for consistency with American Academy of Pediatrics (AAP) guidelines related to infant feeding, sleep, screen time, and safety. Additionally, all unique posts that contained practices inconsistent with these AAP guidelines were thematically coded.

**Results:**

In total, 215 posts were coded. Participants posted 61 questions related to infant health, most commonly solid food introduction (8/61), teething (8/61), and breastfeeding (7/61). Of the 77 answers given by peers, 6 contradicted guidelines. Separately, mothers had 73 posts demonstrating practices inconsistent with AAP guidelines [safe sleep (43/73) and screen time (21/73)].

**Conclusions:**

Mothers’ Facebook group interactions in the context of an infant care intervention revealed that when mothers posed direct questions regarding infant health, their peers generally gave answers that did not contradict AAP guidelines. In contrast, mothers’ posts simply describing sleep and screen time practices commonly contradicted guidelines.

## Background

Social media is a highly prevalent form of communication, used by nearly two thirds of American adults [1]. Parents use social media at even higher rates than the general population, to seek advice, share experiences, and receive social support on parenting-related issues [2], For mothers, whose new role may limit free time [3], social media can be an important and accessible means of communication and social support. In a study of Facebook use during the transition to new motherhood, most women logged into Facebook daily and many women reported increasing their Facebook use after having a baby [4]. In addition to using social media to connect with their existing networks, mothers of infants join social media groups on a variety of parenting topics including breastfeeding [5], prematurity [6], and new motherhood [7].

Through the growing use of smartphones, the “digital divide” of Internet access based on socioeconomic status has narrowed [8]. Low-income new mothers frequently use Internet sources, including social media sites (i.e. Facebook, twitter), to find health and parenting information during the newborn period. In addition, social media may be a particularly useful source of information for low-income mothers as they have have more unmet health information needs in the newborn period compared to higher-income peers [9].

Despite the high prevalence of social media use among parents of all income levels, little is known about the content and quality of parenting and health information shared in these networks and whether this information is in keeping with expert pediatric recommendations. Prior research on social media parenting groups has focused primarily on the social support [6, 7, 10–12] that such groups can provide. To our knowledge, this is the first study assessing the accuracy of the health content shared in a social media parenting peer group. This study is focused on lower-income women; however, it is unknown if higher-income new mothers differ in their social media posts since the literature is limited on this topic. Regardless of the mother’s income status, knowing the accuracy of the information shared is important. As clinical practices integrate the use of technology, informational posts or moderated peer groups through social media platforms may offer opportunities to address misinformation.

Also motivating the present study, the accuracy of pediatric health information in internet sources other than social media has been found to be highly variable [13–18]. For example, one study reviewed 1300 websites with information on infant sleep and found that 28% provided recommendations contrary to American Academy of Pediatrics (AAP) recommendations on safe sleep [15]. In addition to guideline-inconsistent information on infant care from internet sources, family members of infants are reported to commonly give mothers advice contradicting recommendations about sleep, breastfeeding, immunizations, and pacifier use [19].

This study examines the content of health and infant care information shared in a social media parenting group for low-income mothers. After the birth of a baby, mothers often face new pressures as they adapt to their role and acquire new skills. Social media groups offer a unique window into the lives of mothers and are a means of observing how pediatricians’ guidelines may or may not be discussed or practiced in the community. These observations may help pediatricians offer more practical and actionable guidance and support for mothers during this phase of transition.

The objectives of the present study are to: (1) identify the types of health questions asked by low-income mothers of infants in a social media parenting group; (2) describe whether peer answers are consistent with or contradict AAP guidelines; (3) identify the practices that mothers post about that are inconsistent with AAP guidelines.

## Methods

### Study participants

Study participants included the forty-three mothers from the intervention arm of the randomized controlled trial testing Grow2Gether, a peer-based Facebook group intervention aimed at promoting healthy growth in infancy. The forty-four mothers randomized to the Grow2Gether control group of text message appointment reminders were not included in the present study because they did not participate in the Facebook groups. Details of the Grow2Gether intervention have been previously described [20, 21]. Participating mothers were recruited to Grow2Gether at their obstetrics appointments between the 20th and 34th week of pregnancy. To be eligible, these mothers were: 1) receiving prenatal care from a Hospital of the University of Pennsylvania obstetrics practice; 2) age 18 years and older; 3) able to provide informed consent; 4) able to speak, read and write in English; 5) enrolled in Medicaid; 6) overweight or obese; 7) planning for their child to receive primary care services at Children’s Hospital of Philadelphia (CHOP); 9) in possession of a smartphone with both a data and text plan and 10) able to use their phone to obtain photographs and videos prior to enrollment. Any pregnant woman with a major comorbidity (medical or psychological) or pregnant with multiples was excluded.

At 2 months pre-partum, participants were placed into one of four different Facebook groups based on due date. As previously described [20, 21], the private Facebook groups were moderated by a psychologist who was an African American mother of young children. All groups included the same weekly video-based curriculum that was based on AAP Bright Futures guidelines and addressed four main topics: 1) infant feeding practices, 2) sleep, 3) positive parenting, and 4) maternal well-being. The weekly video-based curriculum was designed based on intervention development interviews with 29 mothers and focus groups with 30 pediatric providers. The mothers who participated in the intervention development interviews were recruited from the same geographic region and were socio-demographically similar to the mothers who ultimately participated in the intervention. The pediatric clinicians practiced at pediatric primary care centers that served this same population. The videos were approximately 3–5 min long and featured mothers and infants (many from the same community as the participants) discussing the curriculum content and modeling relevant behaviors [20]. The moderator posted the videos and a brief written summary of the video content, asked discussion questions, and encouraged participants to share experiences and ask questions about their infants. The moderator checked the group several times a day. In order to ensure nothing inappropriate or offensive was posted, the moderator had to approve all posts before they were posted to the group. Throughout the study period, there was only one post not approved as written. We do not have an accurate timestamp to calculate how long it took the moderator to respond to mother’s questions. However, as a rule, the moderator encouraged participants to answer each others questions before posting herself. Mothers were required to post in the group at least once in order to receive their first incentive payment. After the first post, it was suggested that they log in at least weekly to the Facebook group, but there were no additional requirements regarding how to use the group. At enrollment, all participants completed a sociodemographic survey. The Facebook intervention lasted for 11 months, beginning at 2 months pre-partum and continuing until 9 months post-partum. The study was approved by the Children’s Hospital of Philadelphia Institutional Review Board.

### Data analysis

All posts, defined as a wall post, photo or comment, by mothers from 2 months pre-partum to 9 months post-partum were imported into QSR NVivo 10 software (QSR, Burlington, MA). For analysis of the questions that mothers posted about their infants’ health or development, two members of the research team (SK and RG) independently coded all posted questions and iteratively developed the coding scheme. All questions were coded by health topic (Table 2). For each topic, the most recent relevant AAP recommendation that was current during the study period of 2014–2015 was identified. This included AAP Clinical Practice Guidelines, AAP Policy Statements, AAP committee publications, or Bright Futures guidelines [22]. In the case of guidelines for oral teething gels, for which AAP News [23] references FDA guidelines but there is no specific AAP Policy Statement, the FDA warning [24] and the corresponding recommendations from the American Academy of Pediatric Dentistry were used [25]. Peer answers to questions were then coded for consistency with AAP recommendations. Posts by the moderator, including any responses to participant questions, were not coded and were not included in the analysis. Answers that were neither consistent nor in contradiction with AAP recommendations were coded as neutral while answers that directly contradicted or aligned with AAP recommendations were coded as such. All posts were also categorized by whether they were a comment on a moderator’s post, a comment on another participant’s post, or were a unique wall post. For all analyses, discrepancies in coding were discussed until a consensus was achieved.

Separately, to analyze whether mothers’ posts aligned with AAP recommendations, two independent coders (SK and RG) reviewed all other posts (posts that were not questions or responses to other participants’ questions) to determine if they demonstrated practices or beliefs inconsistent with AAP recommendations (Table 3). The recommendation-inconsistent practices were then further categorized by health/safety topic (e.g., sleep position, solid food introduction, walkers). For example, a photo posted of an infant sleeping in the prone position would be coded as a recommendation-inconsistent practice about sleep position since this practice is in contradiction with current AAP back to sleep recommendations [26].

## Results

### Study population

A total of 43 mothers with an average age of 26 years participated in the Grow2Gether Facebook groups (Table 1). Reflecting the study’s focus on low-income mothers, 60% had an annual household income of less than $10,000, 26% were food insecure (as measured by a validated two-item food insecurity screener [27]), and 63% were high school graduates or had less education. Eighty-six percent of participants were Black and 67% had limited health literacy.

**Table 1:** Participant characteristics

Age, y, mean (SD)	25.8 (5.2)
Race and ethnicity, *n* (%)^a^	
Black/African American	37 (86)
White	3 (7)
Hispanic/Latina	2 (5)
Other	3 (7)
Education, *n* (%)	
High school graduate or less	27 (63)
Some college/Associates Degree	14 (33)
Bachelor’s degree or higher	2 (5)
Employment status, *n* (%)	
Working outside the home	18 (42)
Self-employed	2 (5)
Stay-at-home parent	9 (21)
Unemployed	14 (33)
Annual household income, *n (%)*	
Less than $10,000	25 (60%)
$10,000 - $14,999	8 (19%)
$15,000 - $24,999	4 (10%)
≥ $25,000	5 (12%)
Household food security, *n* (%)	
Food secure	32 (74)
Food insecure	11 (26)
Baseline health literacy	
Adequate health literacy	14 (33%)
Possibility/likelihood of limited literacy	29 (67%)

^a^Participants were able to select more than one race and ethnicity

### Health questions and answers

Mothers frequently asked questions in their groups. Specifically, mothers asked 61 questions focused on infant health and development (Table 2). Of the 61 health questions asked, 13 were comments to a post by the moderator, the remainder were either a comment on a post by another participant or were unprompted. The most frequent topics asked about were solid food introduction (8/61), teething (8/61), and breastfeeding (7/61). Overall, feeding (including breastfeeding, solid feeding, formula use) was by far the most frequently asked about topic, accounting for over one-third of mothers’ questions. For example, one mother posted, “is anyone else’s baby greedy...I feel like my son wants to eat every 30 minutes...Help Me.” Another mother asked the group, “I plan to breast feed but what is a good age to stop at?” On the topic of teething, one mother asked “question for the group. It’s about teething. Any recommendations on what to do. I tried teethers and I tried baby oragel and nothing seems to work. These last few nights she has been screaming and it hurts there is nothing I can do to make it stop.”

**Table 2:** Health questions and answers, and consistency with AAP Recommendations

Health Topic	Questions	Contradictory Answers	Neutral Answers	Total Answers^a^
Solid Food Introduction	8	3	4	7
Teething	8	1	4	5
Breastfeeding	7	1	11	12
Rash	5	0	10	10
Infant Behavior	4	0	7	7
Ear Piercing	3	0	9	9
Formula	3	0	2	2
Developmental Milestones	3	0	1	1
Sleep	3	0	4	4
Feeding, other	3	0	7	7
Fever	2	0	2	2
Outdoors	2	0	0	0
Stooling	2	0	1	1
Coughs & Colds	2	1	2	3
Gas & burping	2	0	3	3
Bathing	1	0	1	1
Circumcision	1	0	1	1
Growth	1	0	1	1
Pacifier	1	0	0	0
Vaccines	1	0	1	1

^a^No responses were consistent with AAP recommendations

The vast majority of questions received at least one response and participants posted a total of 77 answers to each other’s questions. Over 90% of the answers were neutral (i.e. neither directly aligned with nor in contradiction with) AAP guidelines. For example, when one mother asked a question about teething, another replied “my baby is doing the same. I bought him a teething ring. He loves it and chews on it like it’s food.” No participant answers directly aligned with AAP guidelines. Six participant answers contradicted AAP guidelines and, of those, half (3/6) were on the topic of introducing solid foods. For example, when one mother asked for tips on getting her infant to eat solids, another mother replied “someone told me mix the baby food inside the bottle.”

### Parent practices inconsistent with expert recommendations

Participants posted 73 instances of advice or images inconsistent with AAP and Bright Futures guidelines (Table 3) on a variety of topics. Of the 73 guideline inconsistent practices, 20 were comments to a post by the moderator; the remainder were either comments to a post by another participant or were unprompted. Unsafe sleep practices and screen time were the topics with the most recommendation-inconsistent posts. Fifty-three percent of the recommendation-inconsistent practices were related to unsafe sleep practices, with posts demonstrating co-sleeping, prone sleep positioning, or unsafe sleep environments. For example, one mother posted a photo depicting her infant sleeping prone while co-sleeping with her sibling.

**Table 3:** Practices Inconsistent with AAP Recommendations

Health Topic	Number of Posts	AAP Recommendation	Source
Sleep environment	24	“Keep soft objects and loose bedding out of crib”	AAP Policy Statement: SIDS and Other Sleep-Related Infant Deaths: Expansion of Recommendations for a Safe Infant Sleeping Environment [26]
Screen time	21	“Discourages media use in children younger than 2 years”	AAP Policy Statement: Media Use in Children Younger Than 2 Years [34]
Co-sleeping	9	“Room-sharing without bed-sharing”	AAP Policy Statement: SIDS and Other Sleep-Related Infant Deaths: Expansion of Recommendations for a Safe Infant Sleeping Environment [26]
Sleep position	6	“Back to sleep for every sleep”	AAP Policy Statement: SIDS and Other Sleep-Related Infant Deaths: Expansion of Recommendations for a Safe Infant Sleeping Environment[26]
Walkers	5	The AAP “recommends a ban on the manufacture and sale of mobile infant walkers.”	AAP Policy Statement Injuries Associated with Infant Walkers [50]
Teething gels	4	“Parents should not use medicated gels to treat teething pain in young children”	AAP News: Baby teething gels not recommended [23]
			FDA Drug Safety Communication [24]
			American Academy of Pediatric Dentistry: Guideline on Infant Oral Health Care [25]
Solid food introduction	3	Solid food introduction between “4–6 months of age”	AAP Committee on Nutrition Pediatric Nutritio Handbook [51]
Juice	1	“Juice should not be introduced into the diet of infants before 6 months of age”	AAP Committee on Nutrition: The Use and Misuse of of Fruit Juice in Pediatrics [52]

Participants also commonly posted photos or descriptions of their infants enjoying screen time. One mother asked the group “is anybody else baby into TV? [My baby] loves Elmo!!!” Similarly, when asked about her daily routine with her baby, another mother posted “[my baby] sleeps thru the night and wakes up early morning like 7:30-8 for a change and a warm bottle and a Lil tv.”

## Discussion

The Grow2Gether Facebook group intervention offered a unique means of observing the concerns, beliefs, and practices of low-income mothers of new infants. Though prior work has described low-income mothers’ self-reported internet and social media use [28] and the social support received from such groups [6, 7, 10–12], this is the first study that examines the content and accuracy of information shared in a social media parenting group of low-income mothers. Prior work has shown that mothers considered the Grow2Gether Facebook group a supportive environment and mothers actively engaged in the group, posting an average of 30 times per week [20, 21]. Mothers were eager to both ask and answer infant health questions in this setting. Answers given to other participants’ questions generally neither endorsed nor contradicted AAP recommendations. Simultaneously, however, parents commonly posted photos and comments that demonstrated practices or beliefs inconsistent with AAP recommendations.

Two infant-care topics emerged as areas where mothers commonly posted practices inconsistent with expert recommendations. First, though there were relatively few questions about sleep, this was the topic with the most posts and photos demonstrating practices inconsistent with AAP recommendations. Specifically, mothers frequently posted photos of infants co-sleeping, sleeping prone, sleeping on unrecommended sleep surfaces (i.e. adult bed, sofa) or sleeping with unrecommended items (i.e. bumpers, pillows, loose bedding). Our findings are consistent with recent work that used nocturnal video recordings and documented high rates of unsafe infant sleep practices even when families knew their actions were being recorded [29]. Similarly, in our study, participants knew a moderator was reviewing their posts, and the Grow2Gether intervention curriculum included a video that reviewed the AAP safe sleep recommendations. Posts depicting unsafe sleep practices were common even after this video was shared. These observations of unsafe sleep practices are concerning given that the racial and ethnic disparities in sudden unexplained infant deaths (SUIDS) may in part be due to differences in adherence to these safe sleep practices [30–33].

Similarly, mothers did not ask any questions about the appropriate use of screen time or media for their infants. Posts or photos depicting infants using screens were quite common in the Facebook groups despite the AAP recommendation in place at the time of this study, which discouraged screen time for children younger than age 2 years [34]. Of note, this recommendation was recently revised to discourage screen time for children younger than 18 months of age [35]. The frequent posts of infants engaging in screen time are consistent with a prior study that found nearly universal exposure to screens among young children ages 6 months to 4 years [36]. This is important because exposure to screens and media during infancy is associated with sleep disturbances [37] and lower congnitive and language development [38, 39].

Though mothers report high levels of trust in their pediatricians on infant care topics [40], our study demonstrates that, even in the context of an infant care intervention, families often do not put expert recommendations into practice, particularly with regard to safe sleep and media use. Importantly, however, parents were open about sharing infant care practices that were inconsistent with AAP guidelines, even in the context of a clinical research study being implemented by a Children’s Hospital. This study did not assess why mothers in our study did not follow AAP-recommended practices. Parent behaviors and beliefs regarding expert recommendations may reflect a lack of awareness or disagreement with the guideline. Additionally, if mothers do not have social support at home or work, there may be practical difficulties with implementing guideline-based parenting practices. In addition, the majority of the women in our study are of racial/ethnic minorities; implicit bias of physicians towards minority adults and children has been well documented [41–45]. Healthcare providers have been found to be less likely to discuss important perinatal health issues, like breastfeeding, with African-American mothers [46] and maternity care practices supporting these behaviors may be lacking in regions with higher numbers of African Americans [47]. Inconsistent healthcare provider reinforcement of AAP endorsed behaviors may have contributed to recommendation-inconsistent practices. Lastly, pediatricians are not the only source of advice on infant care practices, and families may receive conflicting advice from other sources [19].

Further research on the barriers to implementing AAP recommendations regarding safe sleep and screen time may allow pediatricians and others to deliver messages that are more readily and consistently implemented by families. For example, in a study about messaging on safe sleep recommendations, families who received messages framed in terms of both suffocation prevention and SIDS prevention were less likely to use soft bedding as opposed to those who received messages about SIDS prevention alone [48]. Moreover, social media platforms utilized in clinical settings may offer pediatric clinicians an opportunity to identify and discuss problematic infant care behaviors and practices with parents. The use of social media by diverse groups of parents is well-documented [1]. Future research on social media interventions should ensure the adoption of healthy behaviors for women of racial and ethnic minorities as well as those from other groups. In addition, efforts are needed to ensure that supportive groups are available to new mothers, regardless of background.

This study has several limitations. First, the Grow2Gether curriculum, which included content on safe sleep, screen time, and feeding, may have influenced mothers’ beliefs and practices to be more consistent with AAP recommendations, and it is not known what their practices would have been without this intervention. Second, although we know mothers actively engaged and posted in the group, we do not have the means of assessing specifically whether mothers viewed the video curriculum. Third, the presence of the group moderator may have resulted in social desirability bias, and participants may have been hesitant to post photos demonstrating practices or questions contradicting the group curriculum. Still, many guideline-inconsistent posts and practices were recorded despite the Grow2Gether curriculum and presence of a moderator. Fourth, though our sample size was large enough to achieve thematic saturation, the sample was drawn from an infant care intervention for parents from one urban area, so results may not be generalizable to other settings. Fifth, the data examined only the information participants chose to share on social media; we do not know the content of the advice given or practices followed outside of social media. However, prior research suggests that social media posts can be predictive of behavior. [49] Sixth, since there was a small sample size of first time mothers (*n* = 8, out of 43 total), thematic saturation was not reach for this group and we were unable to draw conclusions about how first time versus multiparous mothers used the Facebook group. Seventh, we do not know what other Facebook or social media parenting groups participants may have been members of and the information shared in those channels. Last, since the focus of the present study was on interpreting posts in keeping with professional guidelines, we did not review the codes and themes with participant mothers. Development of future work with social media would benefit from validation by study participants.

## Conclusions

The Grow2Gether Facebook intervention offered a unique window into health-related beliefs, practices, and information sharing among low-income mothers. We found that mothers’ responses to their peers’ questions about infant health generally did not contradict recommendations. However, safe sleep practices and media exposure emerged as prominent areas where mothers commonly did not follow AAP-recommended practices. Future research is needed to explore the barriers to implementing expert recommendations, bolster pediatricians’ messaging on these topics, and potentially enable low-income parents whose children are at higher risk of poor health outcomes to support one another with evidence-based information.

## Abbreviations

AAP: American Academy of Pediatrics
SIDS: Sudden infant death syndrome
SUID: Sudden unexplained infant death
